# Prediction of Aspiration Risk by Using Vocal Biomarkers: Machine Learning Development and Validation Study

**DOI:** 10.2196/86069

**Published:** 2026-03-04

**Authors:** Cyril Varghese, Jianwei Zhang, Sara Charney, Abdelmohaymin A Abdalla, Elizabeth Reeves, Stacy Holyfield, Adam E Brown, Michelle K Higgins, Hunter Stearns, Julie Liss, Nan Zhang, Diana Orbelo, Rebecca L Pittelko, Lindsay Rigelman, Victor Ortega, David G Lott, Visar Berisha

**Affiliations:** 1 Division of Pulmonary and Department of Critical Care Medicine Mayo Clinic in Arizona Phoenix, AZ United States; 2 College of Health Solutions College of Engineering Arizona State University Tempe, AZ United States; 3 Division of Laryngology and Department of Otolaryngology- Head & Neck Surgery Mayo Clinic in Arizona Phoenix, AZ United States; 4 Mayo Clinic Alix School of Medicine Mayo Clinic in Arizona Phoenix, AZ United States; 5 College of Health Solutions Arizona State University Tempe, AZ United States; 6 Department of Quantitative Health Sciences Mayo Clinic in Arizona Phoenix, AZ United States; 7 Division of Laryngology and Department of Otolaryngology- Head & Neck Surgery Mayo Clinic Rochester, MN United States; 8 Division of Pulmonary Mayo Clinic in Arizona Phoenix, AZ United States

**Keywords:** aspiration, voice, speech, artificial intelligence, ARDS, acute respiratory distress syndrome, lung transplant, ILD, interstitial lung disease

## Abstract

**Background:**

Aspiration causes or aggravates a variety of respiratory diseases. Subjective bedside evaluations of aspiration are limited by poor interrater and intrarater reliability, while gold standard diagnostic tests for aspiration, such as video fluoroscopic swallow study and fiberoptic endoscopic evaluation of swallowing, are cumbersome or invasive and health care resource-intensive.

**Objective:**

This study aims to develop and validate a novel machine learning (ML) algorithm that can analyze simple vowel phonations to aid in predicting aspiration risk.

**Methods:**

Recorded [i] phonations during routine nasal endoscopy from 163 unique patients were retrospectively analyzed for acoustic features, including pitch, jitter, shimmer, harmonic to noise ratio, and others. Supervised ML was performed on the vowel phonations of those at high-risk for aspiration versus those at low-risk for aspiration. Ground truth of aspiration risk classification for model development was established using a video fluoroscopic swallow study. The performance of the ML model was tested on an independent, external cohort of patient voice samples. The performance of trained speech language pathologists to categorize high versus low-risk aspirators by listening to phonations was compared against the ML model.

**Results:**

Mean ML risk score for those with the ground truth of high versus low aspiration risk was 0.530 (SD 0.310) vs 0.243 (SD 0.249), which was a significant difference (0.287, 95% CI 0.192-0.381; *P*<.001). In the development cohort, the model showed an area under the curve for the receiver operator characteristic of 0.76 (0.67-0.84) with specificity of 0.76 and *F*_1_-score of 0.63. The performance of the model in an external testing cohort was comparable, with an area under the curve of 0.70 (0.52-0.88), a specificity of 0.81, and an *F*_1_-score of 0.67. The ML model had comparable accuracy, sensitivity, specificity, negative, and positive predictive values compared to trained speech language pathologists in classifying aspiration risk by evaluating vowel phonations.

**Conclusions:**

Otolaryngology (ear, nose, and throat) patients at high risk for aspiration have quantifiable voice characteristics that significantly differ from those who are at a low risk for aspiration, as detected by an ML model trained to analyze sustained phonation and tested on an independent cohort.

## Introduction

Abnormalities of the oropharynx or larynx can cause clinically relevant aspiration of oropharyngeal or gastrointestinal contents into the lungs [1]. Such abnormalities include tumor bulk, resection or radiation injury, pathological or age-related deterioration of nerves or muscles of the upper airways, esophageal abnormalities, and altered sensorium. Acute aspiration can cause significant injuries leading to pneumonias and acute respiratory distress syndrome [1,2]. Chronic aspiration of gastrointestinal contents can aggravate airway and parenchymal lung diseases, such as chronic lipoid pneumonia, bronchiectasis, obliterative bronchiolitis, refractory asthma, and pulmonary fibrosis [3], and is a major risk factor for transplanted lung rejection [4,5].

Aspiration is common but is often undetected and usually only suspected after significant pulmonary damage has occurred. Despite its high prevalence in ambulatory and hospitalized patients [6-8], proactively detecting aspiration risk to prevent downstream sequelae is challenging. The most widely used hospital-based screening method to rule out anterograde aspiration (aspiration that occurs during swallowing) is nursing or speech-language pathologist (SLP) administered bedside swallowing evaluations (BSEs). BSEs vary across institutions but often include listening for a wet cough or wet voice quality after patients swallow varying amounts of liquid. Due to interrater and intrarater reliability issues inherent with these subjective assessments [9], the sensitivity for BSEs ranges from 27% to 85%, with specificities ranging from 50-80% [10], and poor predictive values [11]. BSE results inform referral decisions for gold standard confirmatory testing, including a video fluoroscopic swallow study (VFSS) [12] and fiberoptic endoscopic evaluation of swallowing (FEES) [13]. The VFSS exposes patients to radiation, requires coordinated participation, and can be challenging if patients are acutely ill, delirious, or have mobility issues, for example, in intensive care units. FEES is an invasive and uncomfortable evaluation where a scope is inserted through the nose and oropharynx to visualize the larynx during swallowing. Both VFSS and FEES are resource-intensive, requiring specialized equipment and the expertise of radiologists, laryngologists, and SLPs for administration and proper interpretation, limiting wide-scale deployment as screening tests. Consequently, there is an unmet need for an objective bedside screening test that is easily administered and provides a valid indication of aspiration risk to support decisions regarding referral for VFSS or FEES.

Aspiration involves contact of gastrointestinal/or oropharyngeal contents with the vocal folds as they move through the airway and into the lungs. While perceptual-acoustic studies of postprandial phonation have shown some limited evidence of acute aspiration immediately after a swallow [14-16], chronic exposure to gastrointestinal or oropharyngeal contents is likely to degrade the mucosal surfaces in ways that manifest as changes to the vocal folds’ vibratory and acoustic characteristics. Exposure to gastrointestinal contents can induce histopathological changes to the vibratory margins of the vocal folds, leading to changes in vocal quality [17]. We therefore anticipate that aspirators will exhibit changes in vocal quality relative to non-aspirators either due to underlying pathophysiology or as a consequence of chronic aspiration. In this study, we evaluated voice samples available from Mayo Clinic’s Otolaryngology clinical practices to develop, validate, and externally test a machine learning (ML) algorithm that analyzes voice to predict aspiration risk. We hypothesize that increased risk of aspiration may be associated with changes in human voice quality that can be detected by ML approaches for the objective prediction of aspiration risk.

## Methods

### Voice Data Collection

Voice recordings collected during laryngoscopy exams at Mayo Clinic Arizona (MCA), Otolaryngology clinics were extracted. All voice samples had been originally recorded during routine clinical workflow, using the lapel microphone (of the Pentax Laryngeal Strobe system) clipped near the clavicle. Endoscopy and VFSS databases from January 2020 to July 2021 were curated to identify patients with VFSS, an endoscopy exam, and voice recordings to identify 332 consecutive patients with both VFSS and voice recordings that were within 1 year of each other. Notably, since this is a retrospective study, voice sample collections were not specifically protocolled as would be done in a prospective study. Three SLPs (15 years combined experience) screened and manually excluded (Audacity 3.1.3) recordings with background noise, overlapping sounds (eg, multiple speakers and machine sounds), or low volume. The samples were discarded if SLPs were unable to reliably tag and label the individual samples, per the protocol below. Robust recordings were analyzed without computerized preprocessing to avoid artifacts from being introduced into the signal analysis pipeline. These samples were exported as .wav files, and PRAAT (version 6.2.15) was used to tag the samples with specific labels, including sustained vowel phonation, cued speech, reading, and spontaneous speech. To maximize the number of participants used for training, only [i] vowel phonations were analyzed, as these were most consistently collected across all patients, during routine clinical practice. Focusing on a single vowel type also reduced variability arising from differences in speech elicitation, dialect, or other idiosyncratic speech characteristics. Reducing variability ensures that observed differences in acoustic features reflect true underlying vocal characteristics rather than differences in speaking style or task conditions. Of 772 voice recording sessions from 332 patients, 283 recordings (with each recording containing one to several sustained [i] phonations) from 163 patients were analyzed. An average of 3 clips of the [i] vowel phonations lasting a minimum of 0.5 seconds from the central portion of each recording per patient were used to develop the ML model. These clips were treated as repeated measurements from the same patient rather than independent samples. The patient was defined as the fundamental unit of analysis for both model training and evaluation. All vowel clips from a given patient were always kept together and assigned to the same fold during cross-validation. No clips from the same patient were ever split across training, validation, or test folds.

### Clinical Data Collection

Clinical data extracted included aspiration risk factors, such as upper airway involvement (vocal fold disease, head and/or neck surgery, radiation exposures), esophageal diseases (strictures, impaired motility, GERD), neurological compromise (stroke, neuromuscular, neurodegenerative, or peripheral nerve disease), BMI, and obstructive sleep apnea.

The Rosenbek Penetration-Aspiration Scale (PAS) [18], a well validated and widely used clinical standard [19,20], was used to classify patients VFSS exams as high-risk for aspiration (PAS scores of 6-8, materials into the lower airways); moderate-risk (PAS 3-5 (materials in laryngeal vestibule and/or on vocal folds without being ejecting); and low-risk (PAS 1-2, no penetration or ejected out of airway). PAS measurements are collected as part of routine clinical SLP practice at Mayo Clinic, as is the case in most major medical centers. The moderate aspiration risk group was not included in the test set, as the goal of testing is to observe if this model developed on retrospective voice data can discriminate between high and low risk aspirators. Notably, sustained /i/ phonations were recorded separately from the VFSS on different days. As such, the [i] phonations performed by patients were not influenced by simultaneous VFSS testing.

### Data Preprocessing, ML, and Statistical Analysis

All recordings were processed using a standardized pipeline prior to feature extraction to reduce sensitivity to recording conditions. Audio was converted to mono and resampled to 16 kHz (16-bit pulse-code modulation). A band-pass filter (70-8,000 Hz; 4th-order Butterworth) was applied to reduce low-frequency, handling noise and high-frequency hiss. The retained phonation segment was amplitude-normalized to a fixed level (eg, peak normalization to −1 dBFS [decibels relative to full scale] or root mean square normalization to −20 dBFS). Traditional acoustic features were computed using short-time analysis with 25 ms Hamming windows and 10 ms hop, and summary statistics were aggregated across frames to obtain per-recording feature values.

In our initial model development and training, traditional voice features including pitch, jitter, shimmer, harmonics-to-noise ratio (HNR), cepstral peak prominence (CPP), and relative average perturbation (RAP) were extracted from tagged /i/ samples using audio processing toolboxes PRAAT (GNU General Public License Version 3, 29 June 2007) and Collaborative Voice Analysis Repository for Speech Technologies (COVAREP) [21].

Acoustic features were aggregated at the participant level (by averaging repeated vowel clips) to ensure independence of observations. Group differences between high- and low-risk aspirators were evaluated using 2-sample parametric 2-sided *t* tests. Although the 2-sided *t* test assumes approximate normality, it is generally robust to moderate departures from this assumption when applied to participant-level summaries. Feature distributions were therefore examined for gross irregularities (eg, extreme skewness or outliers), and no violations severe enough to invalidate mean-based comparisons were observed; accordingly, 2-sided *t* tests were used as a univariate screening analysis.

Since multiple acoustic features were evaluated, false discovery rate–adjusted q-values were computed using the Benjamini–Hochberg procedure to account for multiplicity. Raw *P* values are retained in the main text for comparability with prior studies, while the complete set of false discovery rate-adjusted q-values is reported in Multimedia Appendix 1 to guide interpretation under multiple testing.

Comparisons of demographics and clinical characteristics between the groups were performed with the Fisher exact test for categorical variables and the Kruskal-Wallis rank sum test for continuous variables. These analyses were performed with the arsenal package (R4.2.2; R Foundation for Statistical Computing). 0.05 was chosen as the cut-off criterion for statistical significance.

Neural additive models (NAM) [22] were used to classify the voice data and analyze the [i] phonation data of 163 patients. The following dependencies were used for ML coding in Python3: PyTorch, numPy, and sciPy. For NAM, we included 33 features that are well described to have validity in characterizing pathological voice states [23-25].

The patient was defined as the unit of analysis. Five-fold patient-level cross-validation was used for model development and internal validation. All modeling steps, including feature selection, oversampling, hyperparameter tuning, and calibration, were performed exclusively within the training data of each fold, with no information from validation folds used during model fitting or selection.

Recursive feature addition (RFA) [26,27] was nested within the cross-validation framework to avoid optimistic selection bias. Within each training fold, features were added sequentially based on improvement in cross-validated area under the receiver operator characteristic curve (AUC). Feature addition was terminated when performance reached a plateau. To ensure robustness and stability, the final feature subset was defined as the intersection of selected features across cross-validation folds, and validation performance was evaluated using only the feature subset determined from the corresponding training fold.

Hyperparameter tuning for the NAM, including the learning rate and number of training epochs, was conducted using a grid search within each training fold, with performance assessed via an inner validation split.

Class imbalance was addressed during training using random oversampling of the minority class to achieve full class balance, applied only to the training portion of each fold. No oversampling was applied to validation data.

Model calibration was evaluated to assess the reliability of predicted risks. Because uncalibrated NAM outputs exhibited overconfident probability estimates, post-hoc logistic recalibration was applied. Calibration intercept and slope were estimated using predictions from the training folds and then applied to the corresponding held-out validation folds to obtain out-of-sample calibrated probabilities. Calibration performance was summarized using the calibration intercept, calibration slope, and the Brier score.

Model development, internal validation, feature selection, and calibration procedures were conducted and reported in accordance with the TRIPOD-AI (Transparent Reporting of a multivariable Prediction model for Individual Prognosis or Diagnosis-Artificial Intelligence) guidelines for multivariable prediction models using artificial intelligence.

### Independent Model Testing

The development NAM model was trained on voice data from MCA, while independent validation of the model was performed with voice data of patients from another distinct Mayo Clinic site: Mayo Clinic in Rochester. The clinicians, recordings, equipment, rooms, geographic location, and patient demographics of the external testing cohort (Mayo Clinic in Rochester) were independent of the training cohort (MCA). These differences naturally introduced a domain shift, meaning that the distribution of test samples differed from that of the training samples. Evaluating the model under this shift allowed us to assess its out-of-distribution generalizability, ensuring that its predictions reflect aspiration-related signals rather than site-specific confounders. The selection, extraction, tagging, and processing of these externally collected voice samples used the same methodology as described previously for the development cohort. The recording systems in both sites were similar (ie, Pentax systems). ML risk score for each patient in the testing cohort was calculated in a blinded fashion by the model by analyzing [i] phonation clips in the context of the patient’s age and sex. The gold standard designation (ie, aspiration risk category based on VFSS) or clinical information was not known to the investigators running the ML code. Statistical analyses, including sensitivity, specificity, and receiver operator characteristics (ROC), were calculated to assess the model’s performance on both the testing and development cohorts.

### Comparing Human Raters With ML Model

Overall, 4 SLPs, from the 2 medical centers (40 years combined experience), blinded to medical history and aspiration risk, classified patients as at high- or low-risk for aspiration based on perceptual judgements of the [i] phonation clips. This is identical to the information that was provided to the ML model in testing. Notably, the moderate aspiration risk group is not included in the test set, as the goal of testing is to observe if this model developed on retrospective voice data can discriminate between high and low risk aspirators. The interrater reliability for the human raters was assessed using pairwise Cohen kappa. The sensitivity, specificity, positive and negative predictive values, and accuracy of the human raters and the ML model for predicting high versus low-risk aspirators were calculated.

### Ethical Considerations

**Figure 1:**
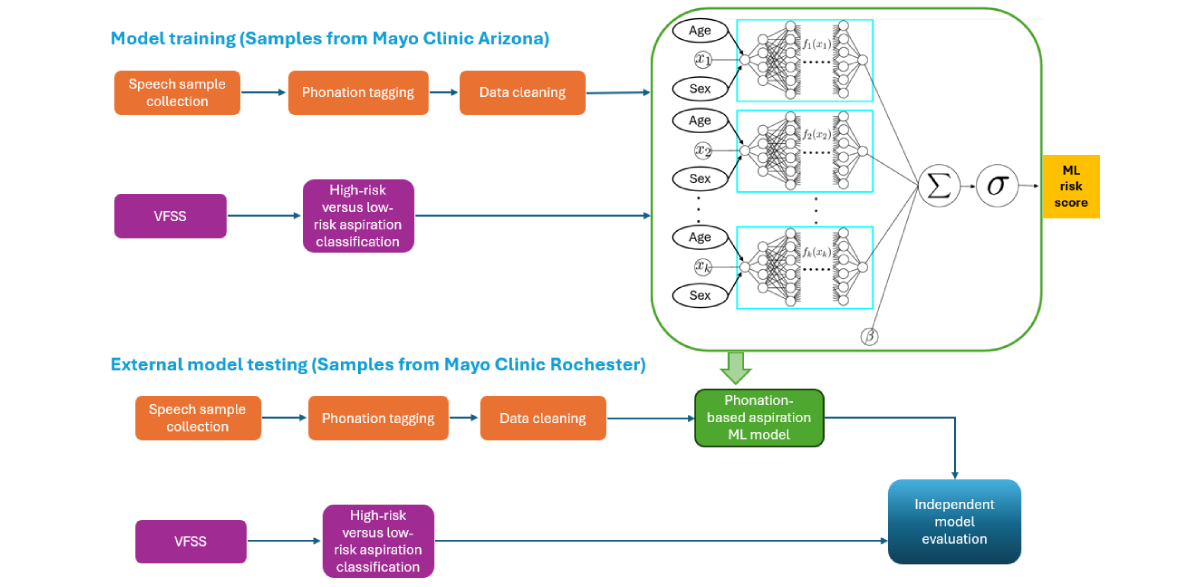
A graphical representation summarizing the workflow for this study from voice sample collection, phonation tagging, and subsequent machine learning model development based on gold standard testing (video fluoroscopic swallow study, determined aspiration risk). Subsequently, independent model evaluation was performed wherein the aspiration risk category based on the video fluoroscopic swallow study was unknown to the model. Contained within the green box is a schematic of the neural additive model [23] trained to estimate risk of aspiration using 33 voice features, in the full cohort (N=163), while adjusting for age and sex, the main confounders between the groups. Σ: summation of all subfeature network outputs and learnable offset β; σ: represent the sigmoid function σ(x)=1/(1+e^(-x) ); β: learnable offset of neural additive model. ML: machine learning; VFSS: video fluoroscopic swallow study.

This study was performed in accordance with the Declaration of Helsinki. This human study was approved by Mayo Clinic Institutional Review Board (approval 21-008975). As part of routine clinical care at Mayo clinic, patients can opt out of having their retrospective clinical data used for research purposes. Patients were not paid for this retrospective study. However, no individual patient’s identifiable information can be found in any part of this manuscript or any of its appendices, ensuring patient privacy. The workflow for voice data curation, ML model development, and validation is shown in Figure 1.

## Results

### Feature Analysis

**Figure 2:**
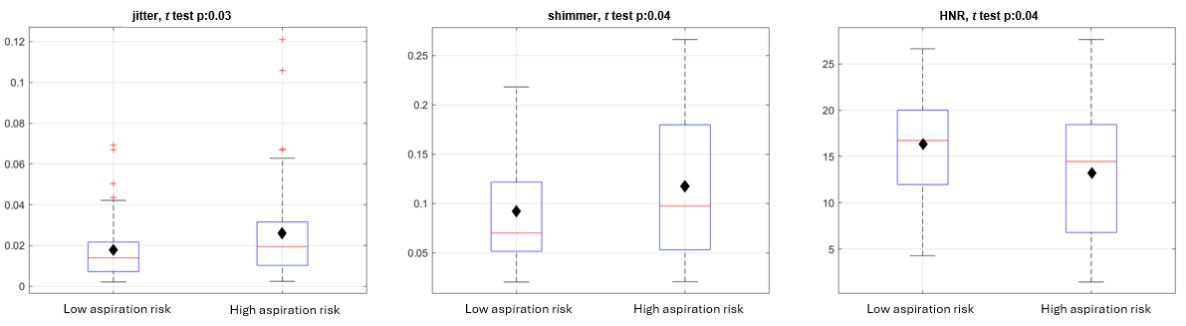
Pilot data of 17 age- and sex-matched aspirators with 17 nonaspirators reveal that aspirators had a higher mean jitter (mean difference 0.0085, 95% CI 0.000756-0.0161), shimmer (mean difference 0.0266, 95% CI 0.00228-0.0506), and lower harmonics-to-noise ratio (mean difference 3.1, 95% CI 0.603-5.6). Means represented by black diamonds. Shimmer is the cycle-to-cycle variability in amplitude, while jitter is the cycle-to-cycle variability in frequency. HNR: harmonics-to-noise ratio.

We initially analyzed retrospective samples from 87 patients (19 high-risk aspirators, 10 medium-risk aspirators, and 58 low-risk aspirators) with an average of approximately 2 voice files per patient. Nonparametric analysis of variance of demographic and clinically relevant risks of aspiration in the pilot data revealed that only age and sex were different among the groups. On age and sex-matching prior to analysis, 17 high-risk aspirators were found to be age- and sex-matched to 17 low-risk aspirators in our exploratory cohort. Of the traditional voice features that were explored, high-risk aspirators were found to have higher jitter and shimmer but lower harmonics richness factor than low-risk aspirators (Figure 2).

### Development of ML Model and Internal Cross-Validation

Our primary ML model development and training cohort evaluated 163 patients within the 3 groups (47 high-risk aspirators, 17 moderate-risk aspirators, and 99 low-risk aspirators) that significantly differed by sex, age, and BMI (with high-risk aspirators having a lower BMI than low-risk aspirators). Additional differences included more reported solid and liquid dysphagia symptoms and a higher frequency of structural changes to head and neck anatomy in the high-risk group than in the low-risk group, as expected in an otolaryngology population (Table 1).

**Table 1:** Baseline characteristics of the training cohort.

Characteristics		High aspiration risk (n=47)	Low aspiration risk (n=99)	Moderate aspiration risk (n=17)	*P* value	
**Sex, n (%)**						<.001^a^
	Male	39 (83)	45 (45.5)	9 (52.9)		
	Female	8 (17)	54 (54.5)	8 (47.1)		
**Age at scope examination**						<.001^b^
	Mean (SD)	72.3 (9.9)	62.9 (12.6)	75.1 (9)		
	Median (IQR)	73 (67-78)	61 (55-72)	77 (65-82)		
	Range	40-88	31-90	62-89		
**BMI (kg/m^2^)**						.009^b^
	Mean (SD)	24.1 (3.2)	27.0 (5.3)	26.4 (7.7)		
	Median (IQR)	23.8 (22-26.1)	26.8 (23.4-29.7)	25.3 (21.3-29.4)		
	Range	18.7-31.2	17-44.2	16-48.9		
**Dysphagia, n (%)**						.06^a^
	No	4 (8.5)	25 (25.25)	2 (11.8)		
	Yes	40 (85.1)	72 (72.7)	14 (82.4)		
	NA^c^	3 (6.4)	2 (2)	1 (5.9)		
**Esophageal disease group, n (%)**						.55^a^
	Clinical GERD^d^	23 (48.9)	46 (46.5)	5 (29.4)		
	Other esophageal disease	3 (6.4)	6 (6.1)	0 (0)		
	Multiple esophageal diseases	2 (4.3)	3 (3)	1 (5.9)		
	GERD on impedance or manometry studies	1 (2.1)	0 (0)	0 (0)		
	No esophageal disease	9 (19.1)	24 (24.2)	4 (23.5)		
	NA	9 (19.1)	20 (20.2)	7 (41.2)		
**OSA^e^, n (%)**						.87^a^
	Compliant with CPAP^f^	8 (17)	19 (19.2)	1 (5.9)		
	Not compliant with CPAP	2 (4.3)	5 (5.1)	1 (5.9)		
	Compliance not reported	2 (4.3)	4 (4)	0 (0)		
	No OSA	35 (74.5)	71 (71.7)	15 (88.2)		
**Neurological illness, n (%)**						.14^a^
	CVA^g^ without any deficit	1 (2.1)	4 (4)	0 (0)		
	ALS^h^	0 (0)	1 (1)	0 (0)		
	Neuromuscular diseases	3 (6.4)	0 (0)	0 (0)		
	CVA with dysphagia only	1 (2.1)	0 (0)	0 (0)		
	CVA with residual neuro deficit	1 (2.1)	0 (0)	0 (0)		
	None	41 (87.2)	94 (94.9)	17 (100)		
**Vocal fold disease, n (%)**						.34^g^
	Yes	23 (48.9)	35 (35.4)	8 (47.1)		
	No	22 (46.8)	62 (62.6)	9 (52.9)		
	NA	2 (4.3)	2 (2)	0 (0)		
**Head and neck anatomical disease, n (%)**						.01^g^
	Surgery	6 (12.8)	13 (13.1)	2 (11.8)		
	Cancer	1 (2.1)	1 (1)	0 (0)		
	Radiation	0 (0)	2 (2)	0 (0)		
	Multiple head and neck anatomical diseases	25 (53.2)	22 (22.2)	5 (29.4)		
	None	15 (31.9)	61 (61.6)	10 (58.8)		

^a^Fisher exact test for count data.

^b^Kruskal-Wallis rank sum test.

^c^NA: not reported by patient or not found in chart review.

^d^GERD: gastroesophageal reflux disease.

^e^OSA: obstructive sleep apnea.

^f^CPAP: continuous positive airway pressure.

^g^CVA: cerebrovascular accident.

^h^ALS: amyotrophic lateral sclerosis.

Baseline characteristics for the age- and sex-matched cohort of 36 high-risk aspirators and 36 low-risk aspirators (N=72) showed that BMI and incidence of head and neck disease were different between the groups (Multimedia Appendix 2).

**Figure 3:**
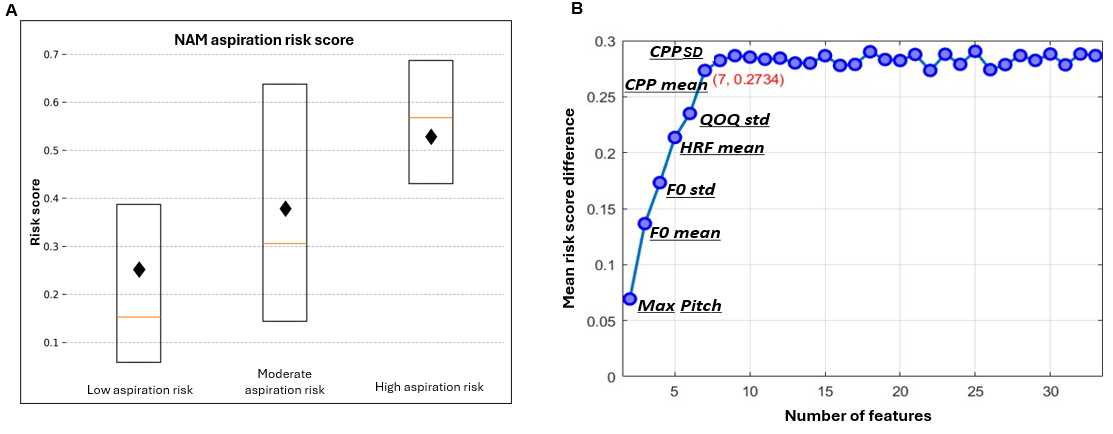
(A) High-risk aspirator neural additive model risk score mean 0.530 (SD 0.31) versus low-risk aspirator risk score of mean 0.243 (SD 0.249); Mean risk difference between groups 0.287 (95% CI 0.192-0.381; *P*<.001). Means represented by black diamonds. Moderate-risk aspirators’ score fell between high- and low-risk aspirators. (B) The recursive feature addition method was used to identify the minimal set of features required to effectively distinguish between high and low-risk aspirators. This method initiates with a single feature and iteratively adds the voice feature that enhances the performance of the existing set. The key metric used to assess the performance of the feature set is the mean risk score difference. CPP: cepstral peak prominence; HRF: harmonic richness factor; NAM: neural additive model; RFA: recursive feature addition.

Because age and sex influence voice, and were different between the groups, they were controlled for in the NAM (Figure 1). The model used 33 extracted features to differentiate between aspirators and controls (Multimedia Appendix 3). Each subnetwork within the model processed a single extracted voice feature along with the corresponding sample sex and age as inputs (Figure 1). The subnetwork output was a single scalar reflecting the adjustments made for age and sex within the feature. The whole NAM output for aspiration risk was a scalar ranging from 0 to 1. High- and low-risk aspirators were clearly distinguished: high-risk aspirator score=0.530 (SD 0.310) versus low-risk aspirator risk score of 0.243 (SD 0.249); mean risk difference between groups was 0.287 (95% CI 0.192-0.381; *P*<.001; Figure 3A). Moderate-risk aspirators had a risk score between high-risk aspirators and low-risk aspirators, although this difference when compared to the other groups was not significant. RFA showed an elbow point at 7 features (Figure 3B), revealing that the most significant voice features adding to the model’s discriminability were the average fundamental frequency and SD (F0_mean; importance=0.45 and F0_std [SD]; 0.29), the maximum fundamental frequency during phonation (Max Pitch; 0.57), the SD of the quai-open quotient that measures the proportion of the glottal cycle when the glottis is open (QOQ_std; 0.04), the average of the harmonic richness factor (HRF_mean; 0.18), the average of the CPP (CPP_mean; 0.07), and the SD of the CPP (CPP_std; 0.04). Feature importance was quantified as the average absolute weights of the corresponding feature-specific subnetworks in the NAM, reflecting each feature’s relative contribution to the model’s overall discriminative performance at a global level.

A description of these features and the other 33 features analyzed in the NAM is in Multimedia Appendix 3. After cross-validated recalibration, the model demonstrated good calibration, with a calibration intercept of –0.06 and a calibration slope of 0.89, indicating minimal global bias and substantially reduced overconfidence. The Brier score improved to 0.189.

### Estimation of Model Performance

**Figure 4:**
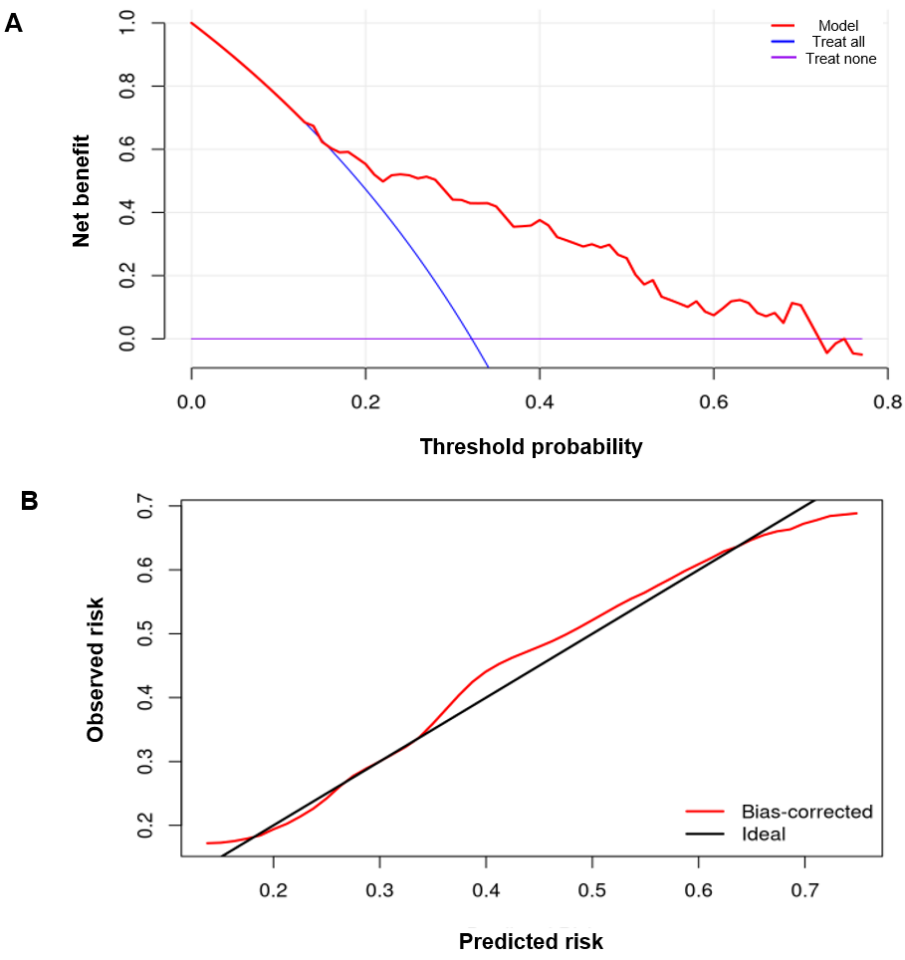
(A) Machine learning risk score to predict aspirators consistently provided higher net benefit than both the “Treat All” and “Treat Non” strategies across a wide range of threshold probabilities (approximately 0 to 0.75). (B) Bootstrap bias-corrected calibration curve closely followed the ideal 45° reference line across the central range of predicted risks.

Decision curve analysis performed on the model showed that using ML risk score for prediction provided more benefit than binary strategies of “Treating all” versus “Treating None”. This was true over a wide range of thresholding probabilities (Figure 4A). Additionally, a bootstrapped bias-corrected calibration analysis showed good agreement between predicted and observed probabilities (Figure 4B).

### External Testing of the ML Model

**Figure 5:**
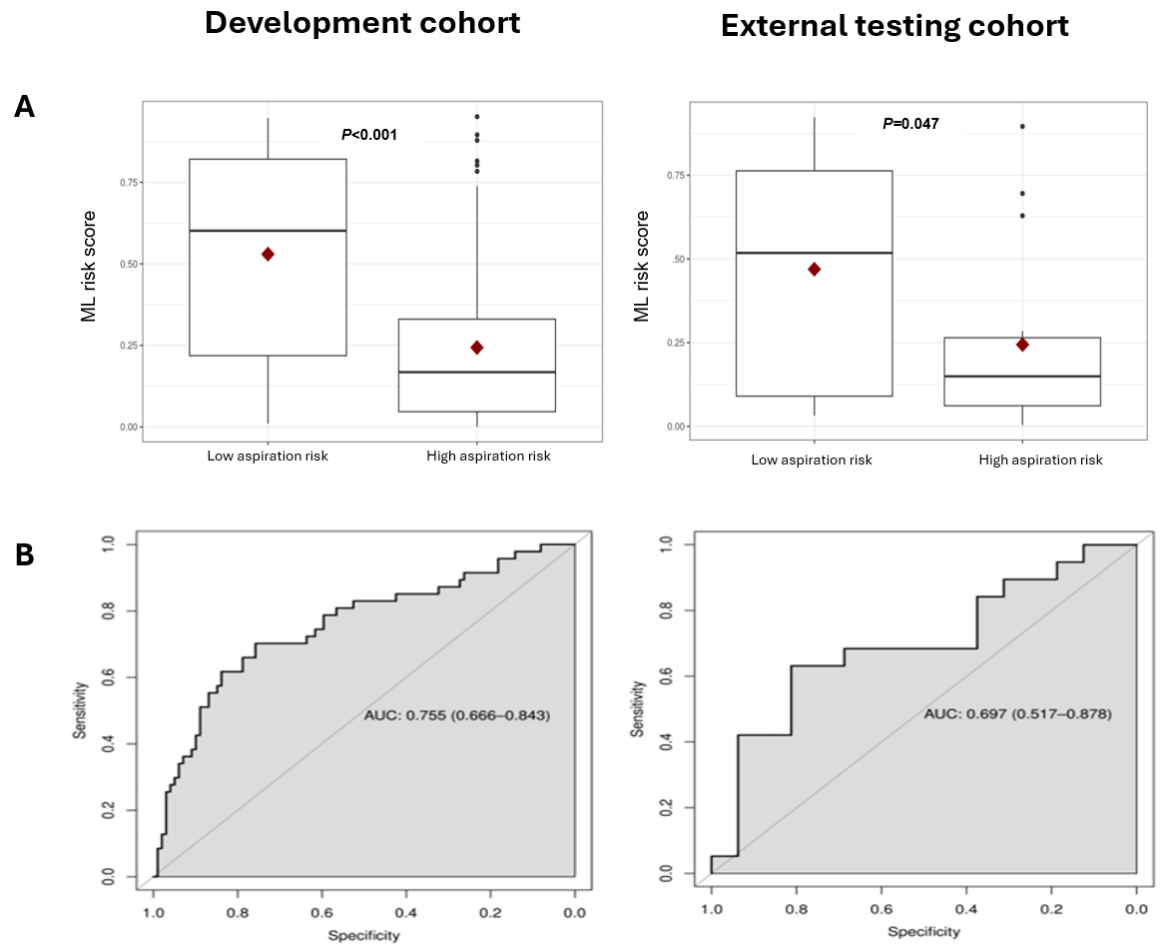
(A) Box plot of machine learning risk score for training versus the external testing cohort. (B) Receiver operator curve for development versus the external testing cohort. AUC: area under the curve; ML: machine learning.

The external cohort contained 19 high- and 16 low-risk aspirators based on VFSS, which totaled 24% of the sample size of high and low-risk aspirators in the development cohort. The mean ML risk score output for the high-risk aspirators was significantly higher than the low-risk aspirators (mean 0.469, SD 0.327 vs mean 0.245, SD 0.265; *P*=.047; Figure 5A). The optimal ML risk score cut-off to distinguish high- from low-risk aspirators by analyzing the training cohort was 0.35 based on the Youden index (which is calculated as sensitivity+specificity–1 and determines the optimal cutoff for balancing sensitivity and specificity). The demographics between the training and testing cohorts were different. (Figure 5B). The AUC for the ROC for the testing cohort was 0.697 (0.517-0.878) compared to the AUC of 0.755 (0.666-0.843) for the training cohort (Figure 5B). The precision, recall, *F*_1_-score, and specificity of the model for the testing cohort were 0.79, 0.57, 0.67, and 0.81, respectively (Table 2). There was not a significant drop in AUC despite the demographic differences between the training and testing cohorts (Table 3). For example, the testing cohort had a higher proportion of female high-risk aspirators (20% vs 5.5%), and a lower proportion of male low-risk aspirators (8.6% vs 30.8%; *P*=.005). The testing cohort was also older (mean age 70.3, SD 13.4 years vs mean 65.9, SD 12.6 years; *P*=.03).

**Table 2:** Analyses evaluating the performance of the model on training versus validation cohort, where F1-score= 2*Precision*Recall/ (Precision + Recall).

Cohorts	Precision	Recall	*F*_1_-score	Specificity
Development	0.58	0.70	0.63	0.76
External testing	0.79	0.58	0.67	0.81

**Table 3:** Illustration of demographic differences between the development and testing cohorts, especially in factors like sex that significantly influence voice.

Aspiration risk		Female	Male
**High, n (%)**			
	Development cohort (%)	8 (5)	39 (26.7)
	Testing cohort	7 (20)	12 (34.3)
**Low, n (%)**			
	Development cohort	54 (37)	45 (30.8)
	Testing cohort	13 (37.1)	3 (8.6)

### Human Raters vs ML Model

Other than 2 raters that had very good agreement (pairwise Cohen Kappa of 0.8), the rest had fair agreement (Cohen κ range 0.34-0.59; Table 4). In classifying patients as a high- or low-risk aspirator by analyzing [i] phonations, the ML model had comparable accuracy to human raters’ range (69% vs 46-60%), sensitivity (58% vs 32-47%) PPV (79% vs 50-78%) and NPV (62% vs 41%-54%; Table 5). The ML model also had comparable specificity to human raters (81% vs 44%-88%). No statistically significant differences were found while comparing the ML model’s performance metrics to the performance of human raters using the McNemar or DeLong test.

**Table 4:** Interrater reliability pairwise κ coefficient between human speech language pathologists’ raters. In parenthesis, interrater reliability between raters is mentioned, as they made predictions of high-risk versus low-risk aspirators.

SLP^a^ raters	Rater 1	Rater 2	Rater 3
Rater 2	0.590 (0.774/0.200)	—^b^	—
Rater 3	0.508 (0.573/0.412)	0.397 (0.573/0.2)	—
Rater 4	0.801 (0.650/1)	0.505 (0.650/0.2)	0.343 (0.249/0.412)

^a^SLP: speech language pathologist.

^b^Not applicable.

**Table 5:** Blinded human (raters 1-4) and machine (rater 5) raters’ ability to predict aspiration risk by listening to phonations against the ground truth (based on video fluoroscopic swallow study).

Reader	Sensitivity, % (95% CI)	Specificity, % (95% CI)	PPV^a^, % (95% CI)	NPV^b^, % (95% CI)	Overall accuracy, % (95% CI)	
Rater 1	36.8 (17.2-61.4)	75 (47.4-91.7%)	63.6 (31.6-87.6)	50 (31.4-68.6)	54.3 (36.9-70.8)	
Rater 2	36.8 (17.2-61.4)	87.5 (60.4-97.8)	77.8 (40.2-96.1)	53.8 (33.7-72.9)	60 (42.2-75.6)	
Rater 3	47.4 (25.2- 70.5)	43.8 (20.8-69.4)	50 (29-71)	41.2 (19.4-66.5)	45.7 (29.2-63.1)	
Rater 4	31.6 (13.6-56.5)	75 (47.4-91.7)	60 (27.4-86.3)	48 (28.3-68.2)	51.4 (34.3-68.3)	
Rater 5 (machine)	57.9 (34-78.9)	81.2 (53.7-95)	78.6 (48.8-94.3)	61.9 (38.7-81)	68.6 (50.6-82.6)	

^a^PPV: positive predictive value.

^b^NPV: negative predictive value.

## Discussion

### Study Strengths

While acutely or chronically having ingested materials enter the larynx alters the quality of voice, human judgment of voice changes [28,29] or even validated tools for perceptual assessments are unreliable screening methods to detect aspiration risk [30,31]. There is a need for tools to objectively quantify aspiration-related voice changes in patients [14-16]. In a study of 93 patients, RAP combined with HNR increased the sensitivity of detecting aspiration in the 5 voice features analyzed [14]. A similar study of 165 patients demonstrated that of 8 voice features evaluated, RAP was the most distinguishing measure between aspirators and non-aspirators [15]. Recently, a study of 198 patients used ML to detect aspiration by analyzing postprandial voice [16]. However, all these studies were single-center studies without an external testing cohort. The focus of these studies was on the effects of anterograde aspiration on voice as ingested materials contact the vocal folds in real time. However, current bedside swallow evaluations also screen for aspiration by making the patient swallow. There are no available tests to objectively detect aspiration risk without having the patient swallow liquids or solids. This can be a limitation for patients who are frail, immobile, and experiencing delirium, who are already at considerable risk for aspiration. An objective screening test for aspiration risk that only requires simple phonation could be useful in these scenarios. Such a method could facilitate frequent longitudinal testing, refining referral for confirmatory testing with VFSS or FEES.

While our model’s performance based on metrics like AUC, *F*_1_-score, and decision curve analysis showed reasonable performance (Figures 4-5), unlike other studies in this space, we also tested the performance of our model in an independent external testing cohort. Previous work has shown that external testing of voice models is critical, as it can be difficult to train clinical speech models that generalize [32-34]. Our testing cohort was from a geographically distinct clinic where retrospective voice samples were collected from patients who were demographically different from our training cohort. Despite these differences, the model’s performance did not change significantly (Tables 2-3 and Figure 5), suggesting that the model is not fragile. Fragile models that are over-fitted in development cohorts tend to perform poorly in external cohorts and therefore can seldom be used effectively in clinical practice. Finally, we explored whether the ML tool added value as a preliminary test to predict aspiration risk by comparing its performance to SLPs. We found that the inter-rater reliability among our SLP raters was only “fair,” similar to reports in the literature for other perceptual evaluations.

There is an inherent tradeoff between purely data-driven model development and approaches that incorporate domain expertise. While deep learning models typically require very large datasets to automatically learn relevant features, simpler supervised ML methods can achieve strong performance with smaller sample sizes when domain experts guide the feature selection and validation process [35]. One of the benefits of NAM used in this evaluation is the sequential way in which features can be considered and confounding variables controlled, thereby improving the explainability of the model [22]. While simpler models like logistic regression can classify by accounting for basic covariates such as age and sex, they are limited in capturing non-linear relationships and feature-specific interactions. The NAM’s feature-specific networks capture complex, nonlinear interactions while controlling for relevant patient characteristics.

**Study Limitations and Future Directions**

This study has several limitations. The relationship between voice and aspiration is likely a complex bidirectional relationship that cannot be fully elucidated with a retrospective analysis. Our population of ear, nose, and throat (ENT) patients has other reasons related or unrelated to aspiration for their voice to be altered (eg, radiation-induced vocal fold scarring). We controlled for age and sex (predictors known to covary with voice) using our NAM. However, other clinical factors like BMI and anatomical head and neck disease are known to covary with aspiration (the response variable) either as a cause or an effect and therefore cannot be easily controlled by generalized regression models in a retrospective analysis. Also, we also focused on anterograde aspiration as confirmed by VFSS. It is possible in our cohorts that participants experienced retrograde aspiration, wherein micro-aspiration of gastrointestinal contents occurs typically during sleep in the context of gastroesophageal reflux disease [3]. Because it can occur in the absence of any structural or functional abnormalities, VFSS or FEES are not sensitive diagnostics. This may have resulted in false positive high-risk errors in both SLP and ML classifications. The inclusion of indirect (reflux symptoms or esophageal impedance testing) and direct indicators of retrograde aspiration (eg, pepsin and bile in bronchoalveolar lavage) in our model may further refine model performance beyond the AUC of .70 range. Additional fine-tuning may be achieved by including measures from connected speech and increasing the diversity of clinical settings. While constrained by the retrospective voice data, this formative study motivates a prospective multi-site voice collection trial to better characterize voice and its relationship to aspiration.

The elbow point in the cross-validation analysis (Figure 3B) was attained at 7 features (relevant features highlighted in Multimedia Appendix 3). These features and interactions were the most predictive of aspiration risk, but only in the specific sample we evaluated, namely ENT patients, while performing the vowel phonation task. We expect that as we expand our databases to include more diverse patient samples from different clinical settings, other voice features may emerge as significant predictors. Our categorization of “high” and “low” aspiration risk was based on VFSS at one point in time. It could be claimed that this is not reflective of the patient's true longitudinal aspiration status. This is why we were careful to label the categories in terms of “risk” rather than absolute diagnoses. Those categorized as high aspiration “risk” were widely separated from the low aspiration “risk” (PAS 6-8 vs PAS 1-2), and crossovers between these categories in our ENT practice, although possible is unlikely. Since PAS scores were obtained from retrospective clinical data and not from a prospective research protocol, interrater reliability of the PAS within our clinical ENT practice cannot be easily calculated. Nevertheless, since the PAS is a well-established and widely used clinical method of estimating aspiration risk, we used this scoring system to define the labels for our cohorts. The performance of the model was only tested for binary discrimination of high-risk versus low-risk aspirators. Aspirators in the moderate group were not tested due to paucity in available retrospective data. However, we recognize that this is an important clinical group that needs to be the focus of future prospective research. It should be noted that SLPs do not make decisions about aspiration risk based on sustained phonations alone. However, this study highlights the potential benefit of ML to complement informed clinical decision-making by experts. Finally, we do not claim that this ML tool can be used as a confirmatory diagnostic test like VFSS or FEES. Rather, the goal of this study was to develop and validate a tool that can potentially serve as an easily deployable screening test used by bedside nurses and SLPs to more objectively screen for anterograde aspiration risk.

### Conclusions

Otolaryngology (ear, nose, and throat) patients at high risk for aspiration have quantifiable voice characteristics that significantly differ from those who are at a low risk for aspiration, as detected by an ML model trained to analyze sustained phonation and tested on an independent cohort. This study uses ML techniques to quantify the quality of voice to estimate aspiration risk without performing a simultaneous swallow evaluation. Future research could include collecting voice samples (including connected speech) from a variety of clinical settings, including intensive care units, hospital wards, and ambulatory clinics, to facilitate model fine-tuning so that it is more generalizable.

## Appendix

Multimedia Appendix 1 Raw *P* values from univariate 2-sided *t* tests comparing acoustic features between high-risk and low-risk aspirators, along with corresponding Benjamini-Hochberg false discovery rate (FDR)-adjusted q-values. Q-values are provided to assess the robustness of univariate screening results under multiple testing and were not used to modify primary analyses or figures.

Multimedia Appendix 2 Baseline characteristics of training cohort when high-risk and low-risk aspirators were age and sex-matched.

Multimedia Appendix 3 List of 33 voice features that were used for machine learning organized in clinically meaningful domains. The seven features that contributed most to the model’s discriminability are bolded. Importance score is the average absolute weights of the corresponding feature-specific subnetworks in the neural additive model, reflecting each feature’s relative contribution to the model’s overall discriminative performance.

## Abbreviations

AUC: area under the curve
CPP: cepstral peak prominence
ENT: ear, nose, and throat
FEES: fiberoptic endoscopic evaluation of swallowing
HNR: harmonic-to-noise ratio
MCA: Mayo Clinic Arizona
ML: machine learning
NAM: neural additive model
PAS: Penetration Aspiration Scale
RAP: relative average perturbation
ROC: receiver operator characteristic
SLP: speech language pathologist
TRIPOD-AI: Transparent Reporting of a multivariable Prediction model for Individual Prognosis Or Diagnosis-Artificial Intelligence
VFSS: video fluoroscopic swallow study
