# Supplementary material for: Prediction of Aspiration Risk by Using Vocal Biomarkers: Machine Learning Development and Validation Study
Source: JMIR Form Res. 2026 Mar 4;10:e86069. doi: 10.2196/86069 (PMC13000375; doi:10.2196/86069)
Supplement: Multimedia Appendix 1 [file formative_v10i1e86069_app1.docx]

**Table Supplemental 1:** Raw p-values from univariate t-tests comparing acoustic features between high-risk and low-risk aspirators, along with corresponding Benjamini-Hochberg false discovery rate (FDR)-adjusted q-values. Q-values are provided to assess the robustness of univariate screening results under multiple testing and were not used to modify primary analyses or figures.

| Feature | p_value | q_value |
| --- | --- | --- |
| jitter_local | 0.0338 | 0.45375 |
| jitter_local_abs | 0.594 | 0.7249 |
| jitter_rap | 0.35 | 0.66495 |
| jitter_ppq5 | 0.245 | 0.66495 |
| jitter_ddp | 0.35 | 0.66495 |
| shimmer_local | 0.041463 | 0.45375 |
| shimmer_local_db | 0.207 | 0.66495 |
| shimmer_apq3 | 0.149 | 0.579333 |
| shimmer_apq5 | 0.373 | 0.66495 |
| shimmer_apq11 | 0.403 | 0.66495 |
| shimmer_dda | 0.149 | 0.579333 |
| mean_F0 | 0.557 | 0.7249 |
| mean_sd | 0.659 | 0.7249 |
| min_pitch | 0.158 | 0.579333 |
| max_pitch | 0.895 | 0.905 |
| mean_hnr | 0.038386 | 0.45375 |
| sd_hnr | 0.13 | 0.579333 |
| peak_slope_mean | 0.389 | 0.66495 |
| peak_slope_std | 0.387 | 0.66495 |
| mdq_mean | 0.59 | 0.7249 |
| mdq_std | 0.28 | 0.66495 |
| naq_mean | 0.528 | 0.7249 |
| naq_std | 0.308 | 0.66495 |
| qoq_mean | 0.619 | 0.7249 |
| qoq_std | 0.643 | 0.7249 |
| h1h2_mean | 0.647 | 0.7249 |
| h1h2_std | 0.503 | 0.7249 |
| hrf_mean | 0.59 | 0.7249 |
| hrf_std | 0.905 | 0.905 |
| psp_mean | 0.242 | 0.66495 |
| psp_std | 0.751 | 0.799452 |
| cpp_mean | 0.055 | 0.45375 |
| cpp_std | 0.117 | 0.579333 |
