# Supplementary material for: Prediction of Aspiration Risk by Using Vocal Biomarkers: Machine Learning Development and Validation Study
Source: JMIR Form Res. 2026 Mar 4;10:e86069. doi: 10.2196/86069 (PMC13000375; doi:10.2196/86069)
Supplement: Multimedia Appendix 2 [file formative_v10i1e86069_app2.docx]

**Table Supplemental 2:** Baseline characteristics of training cohort when high-risk and low-risk aspirators were age and sex-matched.

|  | High Aspiration Risk (N=36) | Low Aspiration Risk (N=36) | p value |
| --- | --- | --- | --- |
| **Sex** |  |  | 1.000^1^ |
| Male | 28 (77.8%) | 28 (77.8%) |  |
| Female | 8 (22.2%) | 8 (22.2%) |  |
| **Age at Scope Exam** |  |  | 0.636^2^ |
| Mean (SD) | 70.9 (10.2) | 70.1 (10.0) |  |
| Median | 73.0 | 71.0 |  |
| Q1, Q3 | 65.8, 77.2 | 66.8, 77.0 |  |
| Range | 40.0 - 88.0 | 40.0 - 90.0 |  |
| **BMI** |  |  | 0.003^2^ |
| Mean (SD) | 24.1 (3.0) | 27.6 (5.5) |  |
| Median | 23.7 | 27.0 |  |
| Q1, Q3 | 22.8, 26.0 | 23.8, 30.2 |  |
| Range | 18.7 - 31.2 | 17.0 - 44.2 |  |
| **Dysphagia** |  |  | 0.045^1^ |
| No | 3 (8.3%) | 9 (25.0%) |  |
| Yes | 30 (83.3%) | 27 (75.0%) |  |
| NA | 3 (8.3%) |  |  |
| **Esophageal disease group** |  |  | 0.888^1^ |
| Clinical GERD | 16 (44.4%) | 14 (38.9%) |  |
| Other esophageal disease | 3 (8.3%) | 3 (8.3%) |  |
| Multiple esophageal diseases | 1 (2.8%) | 1 (2.8%) |  |
| GERD on impedance/manometry studies | 1 (2.8%) | 0 (0.0%) |  |
| No esophageal disease | 7 (19.4%) | 11 (30.6%) |  |
| Not reported | 8 (22.2%) | 7 (19.4%) |  |
| **OSA** |  |  | 0.608^1^ |
| Compliant with CPAP | 6 (16.7%) | 10 (27.8%) |  |
| Not compliant with CPAP | 2 (5.6%) | 1 (2.8%) |  |
| Compliance not reported | 1 (2.8%) | 2 (5.6%) |  |
| No OSA | 27 (75.0%) | 23 (63.9%) |  |
| **Neurological illness** |  |  | 0.186^1^ |
| CVA without any deficit | 0 (0.0%) | 2 (5.6%) |  |
| Neuromuscular diseases | 2 (5.6%) | 0 (0.0%) |  |
| CVA with dysphagia only | 1 (2.8%) | 0 (0.0%) |  |
| neuro not mentioned | 3 (8.3%) | 1 (2.8%) |  |
| None | 30 (83.3%) | 33 (91.7%) |  |
| **Vocal fold disease** |  |  | 0.926^1^ |
| Yes | 17 (47.2%) | 15 (41.7%) |  |
| No | 17 (47.2%) | 19 (52.8%) |  |
| Not reported | 2 (5.5%) | 2 (5.6%) |  |
| **Head and neck anatomical disease** |  |  | 0.056^1^ |
| Surgery | 2 (5.6%) | 3 (8.3%) |  |
| Cancer | 1 (2.8%) | 0 (0.0%) |  |
| Radiation | 0 (0.0%) | 1 (2.8%) |  |
| Multiple head and neck anatomical diseases | 21 (58.3%) | 11 (30.6%) |  |
| None | 12 (33.3%) | 21 (58.3%) |  |

1. Fisher’s Exact Test for Count Data

2. Kruskal-Wallis rank sum test

NA: Not reported by patient or not found on chart review
