# Supplementary material for: Prediction of Aspiration Risk by Using Vocal Biomarkers: Machine Learning Development and Validation Study
Source: JMIR Form Res. 2026 Mar 4;10:e86069. doi: 10.2196/86069 (PMC13000375; doi:10.2196/86069)
Supplement: Multimedia Appendix 3 [file formative_v10i1e86069_app3.docx]

**Table Supplemental 3:** List of 33 voice features that were used for machine learning organized in clinically meaningful domains. The seven features that contributed most to the model’s discriminability are bolded. Importance score is the average absolute weights of the corresponding feature-specific subnetworks in the neural additive model, reflecting each feature’s relative contribution to the model’s overall discriminative performance.

| Group | Feature | Importance  Score | Description |
| --- | --- | --- | --- |
| Jitter Features  These measure variations in pitch and are indicators of vocal stability. Higher values of jitter measurements typically indicate greater variability, which is often associated with pathological voices. | jitter_local | 0.01 | Average absolute difference between consecutive periods, divided by the average period. |
|  | jitter_local_abs | 0.01 | Average absolute difference between consecutive periods, in seconds. |
|  | jitter_rap | 0.02 | Relative average perturbation, the average absolute difference between a period and the average of it and its two neighbors, divided by the average period. |
|  | jitter_ppq5 | 0.02 | Five-point Period Perturbation Quotient, the average absolute difference between a period and the average of it and its four closest neighbors, divided by the average period. |
|  | jitter_ddp | 0.01 | Average absolute difference between consecutive differences between consecutive periods, divided by the average period. |
| Shimmer Features  These measure variations in amplitude of the acoustic signal, reflecting vocal fold vibration regularity. Higher shimmer values suggest more amplitude variability, commonly found in voices with pathologies. | shimmer_local | 0.02 | Average absolute difference between the amplitudes of consecutive periods, divided by the average amplitude. |
|  | shimmer_local_db | 0.03 | Average absolute base-10 logarithm of the difference between the amplitudes of consecutive periods, multiplied by 20. |
|  | shimmer_apq3 | 0.02 | Three-point Amplitude Perturbation Quotient, the average absolute difference between the amplitude of a period and the average of the amplitudes of its neighbors, divided by the average amplitude. |
|  | shimmer_apq5 | 0.02 | Five-point Amplitude Perturbation Quotient, the average absolute difference between the amplitude of a period and the average of the amplitudes of it and its four closest neighbors, divided by the average amplitude. |
|  | shimmer_apq11 | 0.03 | 11-point Amplitude Perturbation Quotient, the average absolute difference between the amplitude of a period and the average of the amplitudes of it and its ten closest neighbors, divided by the average amplitude. |
|  | shimmer_dda | 0.02 | Average absolute difference between consecutive differences between the amplitudes of consecutive periods. |
| Pitch Features | **F0_mean** | **0.45** | **Average fundamental frequency, related to the perceived pitch of the voice.** |
|  | **F0_std** | **0.29** | **Standard deviation of the fundamental frequency, indicating how much pitch varies over time.** |
|  | min_pitch | 0.03 | Minimum fundamental frequency during phonation. |
|  | **max_pitch** | **0.57** | **Maximum fundamental frequency during phonation.** |
| Harmonic Features  These features reflect voice quality (clarity/richness) and can help identify abnormalities or pathologies. | hnr_mean | 0.01 | Mean harmonics-to-noise ratio, indicating the ratio of harmonic sound to noise in the voice signal. |
|  | hnr_std | 0.02 | Standard deviation of the HNR, reflecting variability in voice quality. |
|  | h1h2_mean | 0.03 | Mean difference in amplitude between the first and second harmonics. |
|  | h1h2_std | 0.01 | Standard deviation of the difference between the first and second harmonics. |
|  | **hrf_mean** | **0.18** | **Mean of the harmonic richness factor, a measure of how much energy is in the harmonics relative to the fundamental frequency.** |
|  | hrf_std | 0.02 | Standard deviation of the harmonic richness factor, reflecting variability in harmonic richness. |
| Glottal Source Analysis Features | peak_slope_mean | 0.01 | Average slope of the spectral peaks, indicative of how sharply energy dissipates |
|  | peak_slope_std | 0.01 | Standard deviation of the spectral peak slope, showing variability in how energy distribution changes over time. |
|  | mdq_mean | <0.01 | Average of the Maxima Dispersion Quotient (MDQ), often related to the rate of vocal fold closure. |
|  | mdq_std | 0.02 | Standard deviation of the MDQ, indicating the consistency of vocal fold closure. |
|  | naq_mean | 0.03 | Mean normalized amplitude quotient (NAQ), which relates to the efficiency of the glottal closure during phonation. |
|  | naq_std | 0.03 | Variability in NAQ, indicating how consistently the vocal folds are closing. |
|  | qoq_mean | 0.03 | Average quai-open quotient (QOQ), which measures the proportion of the glottal cycle during which the glottis is open. |
|  | **qoq_std** | **0.10** | **Standard deviation of the QOQ, indicating the consistency of glottal opening during phonation.** |
|  | psp_mean | 0.01 | Average peak slope of the spectrum, offering insight into how spectral energy is concentrated around the peak frequency. |
|  | psp_std | 0.01 | Variability in the spectral peak slope, which can reflect changes in vocal tract dynamics or resonance strategies. |
|  | **cpp_mean** | **0.07** | **Mean cepstral peak prominence, which is a measure of the prominence of the peak in the cepstral domain, indicative of voice quality.** |
|  | **cpp_std** | **0.04** | **Variability in CPP, indicating how consistently the voice can produce clear, strong cepstral peaks.** |
